# Temporomandibular Joint Prosthesis in a Patient with Congenital Infiltrating Lipomatosis of the Face with Bony Ankylosis of the Temporomandibular Joint: A Case Report

**DOI:** 10.3390/jcm12247723

**Published:** 2023-12-16

**Authors:** Lauren C. M. Bulthuis, Jean Pierre T. F. Ho, Petra C. M. Zuurbier, Michail Koutris, Jitske W. Nolte, Jan de Lange

**Affiliations:** 1 Department of Oral and Maxillofacial Surgery, Amsterdam Universitair Medische Centra, University of Amsterdam, Meibergdreef 9, 1105 AZ Amsterdam, The Netherlands; j.p.ho@amsterdamumc.nl (J.P.T.F.H.); j.w.nolte@amsterdamumc.nl (J.W.N.); j.delange@amsterdamumc.nl (J.d.L.); 2 Academic Centre for Dentistry Amsterdam (ACTA), University of Amsterdam and Vrije Universiteit Amsterdam, 1081 LA Amsterdam, The Netherlands; 3 Department of Oral and Maxillofacial Surgery, Northwest Clinics, 1815 JD Alkmaar, The Netherlands; 4 Department of Orthodontics, Academic Centre for Dentistry Amsterdam (ACTA), University of Amsterdam and Vrije Universiteit Amsterdam, 1081 LA Amsterdam, The Netherlands; p.c.m.zuurbier@acta.nl; 5 Department of Oral Kinesiology, Academic Centre for Dentistry Amsterdam (ACTA), University of Amsterdam and Vrije Universiteit Amsterdam, 1081 LA Amsterdam, The Netherlands; m.koutris@acta.nl

**Keywords:** case report, hemifacial hyperplasia, mandibular asymmetry, hemimandibular elongation, maxillofacial surgery

## Abstract

Hemifacial hyperplasia (HFH) is a rare congenital disorder characterized by marked unilateral overgrowth of the facial tissues. A subtype of HFH is congenital infiltrating lipomatosis of the face (CIL-F). This disease is characterized by unilateral diffuse infiltration of mature adipose cells in the facial soft tissue and is associated with skeletal hypertrophy. This work aims to report a case of a CIL-F patient with right facial asymmetry and progressive growth at adolescent age, causing mandibular asymmetry due to signs of concomitant unilateral condylar hyperplasia. At the age of seventeen, a condylectomy was performed to stop the progression of asymmetric mandibular growth. Five years later, the patient developed CIL-F-associated temporomandibular joint ankylosis, manifesting as progressive restricted mouth opening along with temporal facial pain. In this CIL-F patient, a TMJ reconstruction with an alloplastic total joint prosthesis was successfully performed with optimal maximal mouth opening, complete alleviation of temporal facial pain, and stable dental occlusion one year postoperatively. A TMJ reconstruction with a complete alloplastic total joint prosthesis proved to be a predictable, stable, and safe treatment option in a patient with CIL-F-associated TMJ ankylosis who was previously treated with condylectomy due to progressive mandibular asymmetry.

## 1. Introduction

Hemifacial hyperplasia (HFH) is a rare congenital disorder characterized by marked unilateral overgrowth of the hard and soft facial tissues, resulting in a marked asymmetry of the face. In most cases, it is present at birth and shows subsequent proportional growth until the patient reaches the age of adulthood, at which point the growth stops [1].

Congenital infiltrating lipomatosis of the face (CIL-F) is part of the HFH spectrum. It is characterized by unilateral diffuse infiltration of mature adipose cells in the facial soft tissue and is associated with skeletal hypertrophy [2]. Congenital infiltrating lipomatosis (CIL) is a subgroup of lipomatous tumors with an unknown etiology [2]. It was first described by Slavin et al. [3] as a distinct clinico-pathological entity. Patients with CIL-F show diffuse infiltration of facial soft tissue by mature adipose cells, osseous hypertrophy, and a high risk of recurrence after surgery [3]. Osseous tissue changes in CIL-F involve sclerosis and hyperplasia of the cervical vertebrae, skull, hemimandibular hyperplasia (including mandibular body, ramus, and condylar processes), accelerated dentoskeletal growth, and zygomatic hyperplasia [4]. It is usually present at birth or found early after birth [4]. CIL-F may progress rapidly during childhood with extensive hyperplasia or as an indolent form with gradual hyperplasia at the age of adulthood, at which time patients present themselves seeking care [5].

To our knowledge, there have been a few reports [2,6,7] on CIL involving the face, wherein the disease is associated with underlying bony ankylosis of the temporomandibular joint (TMJ). No case of TMJ reconstruction with a complete alloplastic total joint prosthesis in a CIL-F patient has been previously described in the literature. In patients with TMJ ankylosis, a resection of the TMJ and reconstruction with a complete alloplastic total joint prosthesis can reduce the chances of re-ankylosis and might allow for facial asymmetry reduction or correction [8]. Reconstruction of the TMJ can restore the form and function of the joint, reduce pain, and improve the patient’s quality of life [8].

The aim of our study was to report a case of a CIL-F patient with concomitant signs of unilateral condylar hyperplasia (UCH) and associated ankylosis of the TMJ, years after condylectomy, in whom a TMJ reconstruction with a complete alloplastic total joint prosthesis procedure was successfully performed.

## 2. Clinical Presentation

The patient in this paper was previously described in a case report by Nolte et al. [9] about the detection of a somatic mutation in PIK3CA.

The patient was the second-born child of healthy, non-consanguineous parents. There was no history of congenital anomalies or asymmetries in the family. At a young age, the parents noticed a slightly larger right cheek without any further facial abnormalities. The first evaluation took place at the age of 3 because of facial asymmetry. The right half of the patient’s face was more prominent than the left side, with hyperemic spots, and there was an enlargement of the right side of the tongue compared to the left side. The permanent incisors were visible on the right side.

Over the years, the patient developed intraoral excessive soft tissue of the right cheek, referred to as a hamartoma, which was surgically removed at the age of 6 years. Histological analysis revealed the presence of fibrous tissue, muscle, adipose tissue, and a significant abundance of nerve fibers.

**Figure 1:**
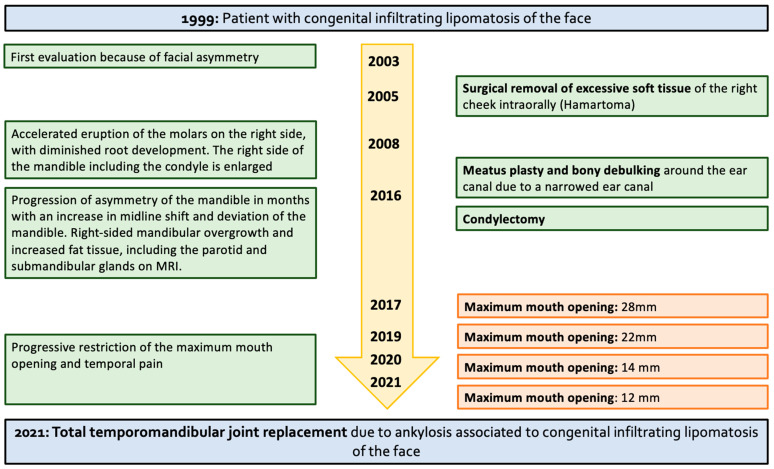
A timeline of the clinical features, diagnostic findings, and surgical management over the years.

From the age of 8 years, panoramic radiographs showed enlargement of the right mandible as well as the condyle and accelerated eruption of the molars with diminished root development on the right side. In the following years, the asymmetry of the patient’s face became more prominent. At the age of 16 years, an intervention was needed due to a narrowed ear canal, and it was urged for meatus plasty and bony debulking around the ear canal. In addition, an adequate dental occlusion with a median position of the upper and lower midline was achieved at the age of 16 with subsequent orthodontic treatment (Figure 1).

**Figure 2:**
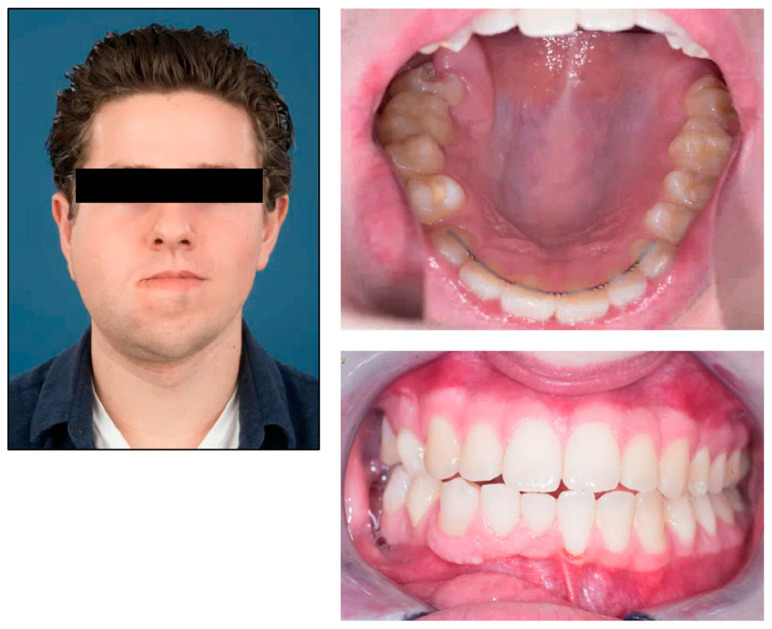
The facial clinical characteristics, occlusal state, and mouth opening at the age of nineteen.

One year later, the patient presented a rapid progression of the asymmetry of the right mandible over the course of months. This enlargement resulted in an increase in midline shift and deviation of the mandible. Magnetic resonance imaging (MRI) was performed and showed overgrowth of the right-sided mandibular and increased fat tissue, including the parotid and submandibular glands. Hyperactivity of the osteoblasts in the right condyle was confirmed with a single-photon emission computed tomography (SPECT-CT). Due to the progressive facial asymmetry, a right-sided condylectomy was performed. Histological examination of the resected condylar tissue displayed cross-sections of trabecular bone tissue with normocellular bone marrow interspersed and a normal proportion of hematopoietic series. The progenitor cells exhibited an organoid appearance without prominent focal concentrations. On the surface, there was a presence of fibrous tissue overlaying a thick layer of hyaline cartilage, with the periosteum beneath it. At follow-up after one year, no progression of mandibular asymmetry was evident (Figure 2).

### 2.1. Clinical Findings and Diagnostic Assessment

Four years later, the patient presented with progressive restriction of the maximum mouth opening (MMO). On intra-oral examination, a bony density was palpable on the right zygoma. A computed tomography (CT) scan showed a minimal increase of the neo-condyle and marked osseous hypertrophy of the coronoid process, forming a neo-articulation with the anterior part of the zygomatic arch, and no radiological signs of osteoma or osteochondroma. After two months, the patient presented with temporal facial pain and a further decrease in mouth opening. Due to the progressive restriction of the mouth opening (12 mm), surgical intervention was indicated. Considering the risk of scar tissue development and new CIL-F overgrowth after gap-arthroplasty, temporomandibular joint reconstruction was chosen as the treatment of choice. The primary purpose of the surgery was to improve the patient’s mouth opening, relieve facial pain, and maintain a stable occlusion.

### 2.2. Surgical Intervention

**Figure 3:**
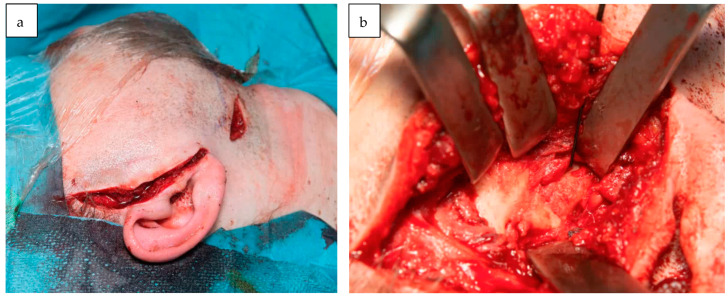
The surgical approach to TMJ reconstruction using a pre-auricular and peri-angular mandibular incision (**a**). An intraoperative image illustrating the partial bony ankylosis of the TMJ (**b**).

**Figure 4:**
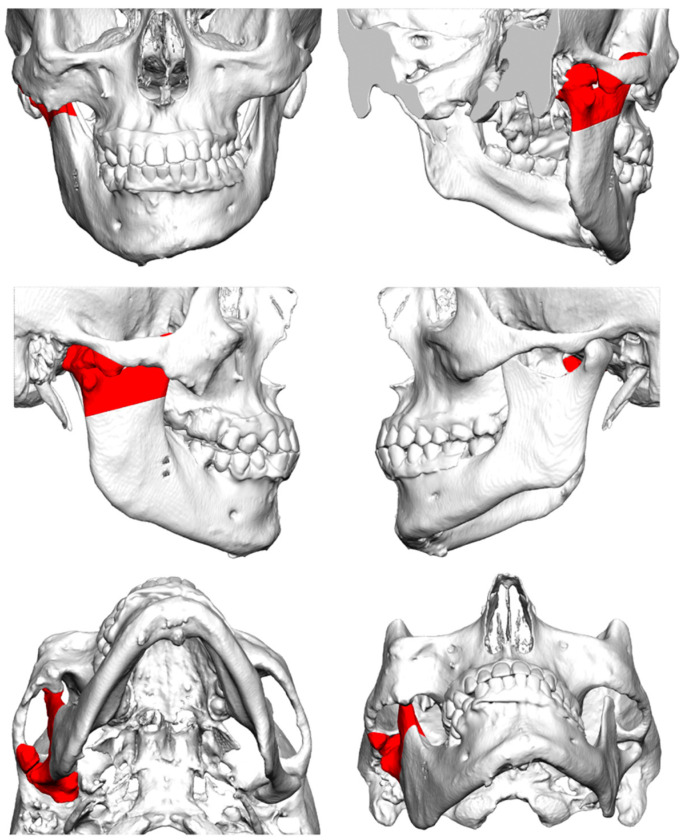
The resected bone (red) of the mandibular fossa, articular tubercle, coronoid process, and condyloid process.

**Figure 5:**
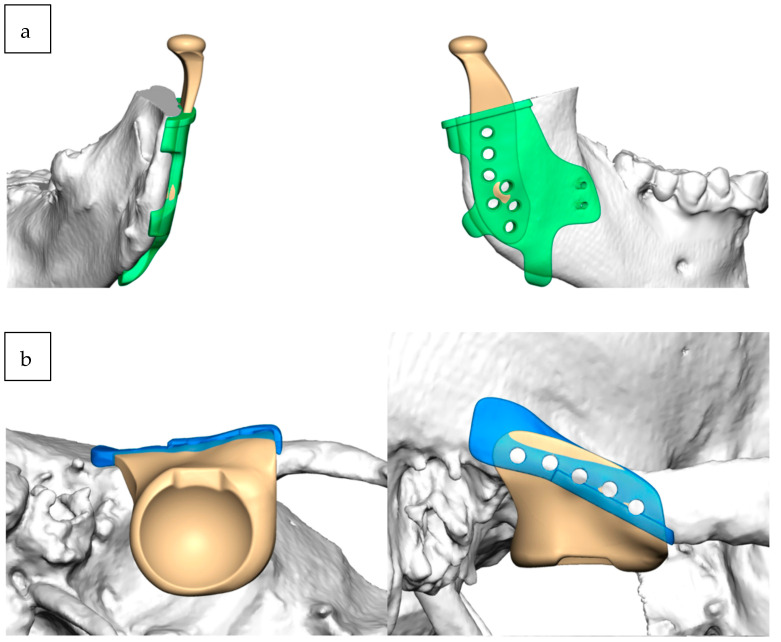
The right mandibular marking guide (green) and prosthesis (**a**) and the right fossa marking guide (blue) and prosthesis (**b**).

Surgical treatment consisted of a pre-auricular incision via the condylectomy scar, followed by a peri-angular mandibular incision and approach (Figure 3) [10]. Soft tissue overgrowth and scar tissue were observed. After obtaining sufficient visibility of the articulation fossa, condyloid, and coronoid processes, the guiding screws were removed, and the resection template was positioned and fixed using the submandibular approach. Resection of the mandibular fossa, articular tubercle, coronoid process, and condyloid process was performed with three-dimensional (3D) computer-aided design and computer-aided manufacturing (CAM/CAM) resection guides (Figure 4) [11]. The patient was placed into temporary intraoperative maxillomandibular fixation with power chains [12], followed by placement and fixation of the patient-specific fossa component and condylar component (Figure 5). Following an infraumbilical incision of 3 cm, subcutaneous adipose tissue was dissected, and a sufficient amount of adipose tissue to cover the prosthesis was harvested. The abdominal fat graft was placed around the prosthesis, and the incisions were sutured [13].

### 2.3. Follow-Up

**Figure 6:**
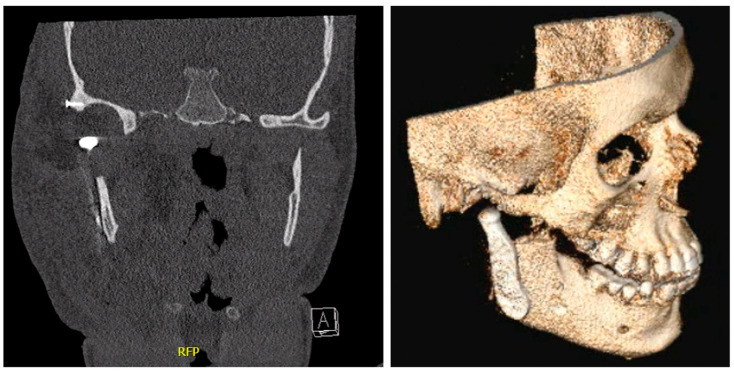
A CT scan and 3D animation one day after right-sided TMJ reconstruction.

**Figure 7:**
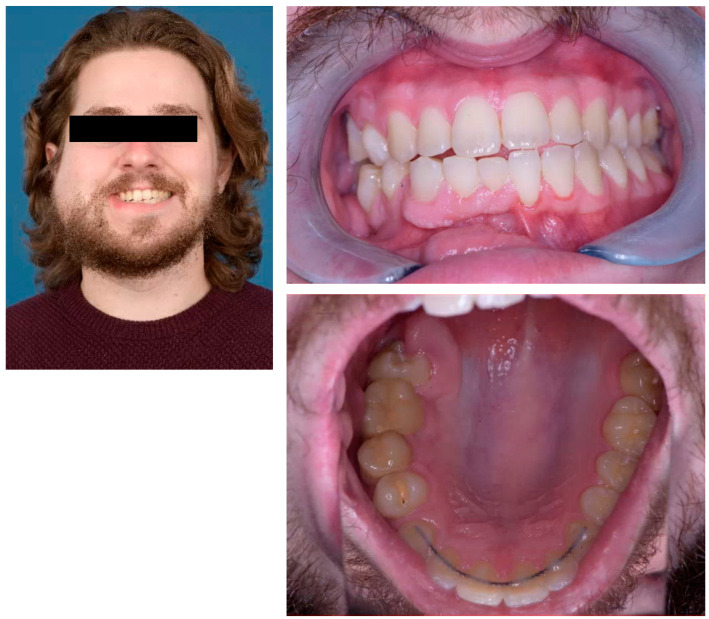
The facial clinical characteristics, occlusal state, and mouth opening three months post-surgery.

The CT scan following one day post-surgery showed the right-sided unilateral overgrowth of the remaining part of the right mandible, with the TMJ prosthesis in situ (Figure 6). Six days after TMJ reconstruction, there was an improvement in the mouth opening with a MMO of 20 mm. One month, three months, four months, and one year after TMJ reconstruction, the MMO was 24 mm, 38 mm, 40 mm, and 41 mm, respectively (Figure 7). One year after surgery, the patient experienced no difficulties with food intake, the temporal facial pain completely receded after four months, and a stable occlusion was present. There were no signs of a postoperative recurrence. In addition to providing restoration of joint function and improvement of pain, the procedure also led to an improvement in the patient’s quality of life.

## 3. Discussion

The clinical features of CIL-F in the HFH spectrum comprise unilateral overgrowth of the hard and soft facial tissues. Osseous tissue changes include hemimandibular hyperplasia and accelerated dentoskeletal growth [4]. The term congenital infiltrating lipomatosis of the face was first described by Slavin et al. [3] in 1983. This study reported three patients with congenital fatty buccal hypertrophy, and they described the following characteristics of the CIL lesion: a nonencapsulated tumor containing mature fat cells, infiltration of adjacent muscle and soft tissue, the absence of malignant characteristics, the absence of lipoblasts, the presence of fibrous elements in conjunction with increased numbers of nerve bundles and vessels, and hypertrophy of adjacent bone [3]. The etiology of CIL-F remains unknown [14]. The prevailing pathogenic hypothesis suggests a somatic mutation occurring in the PIK3CA gene within the affected tissues [15,16]. This mutation is also identified in cancers and affected tissues that are associated with various non-inherited overgrowth disorders. PIK3, encoded by the PIK3CA gene, plays a pivotal role in governing cell proliferation, adhesion, survival, and motility. External environmental factors, such as radiation, trauma, and degenerative processes leading to fatty transformation, have the potential to initiate and expedite the development of lipomatous changes [15]. CIL-F patients present with facial asymmetry from birth, and this asymmetry evolves as the patient matures [14]. Typically, the lesion occupies the lower to middle third of one side of the face unilaterally [14,17]. There are no observed changes in the color or texture of the adjacent skin or mucosa. Early tooth eruptions may also be noted [18]. The primary potential diagnoses that need to be considered include lymphangioma, angiolipoma, hemangioma, parotid tumors, neurofibromatosis, and other tumors characterized by fat infiltration, such as liposarcomas and lipoblastomas [14]. After excluding these potential diagnoses, clinical and histological characteristics enable the diagnosis of congenital hemifacial hypertrophy [4]. A diagnosis of CIL-F is clinically confirmed through imaging studies and histopathologically and can be identified by the presence of hemimandibular hypertrophy and accelerated growth of the dentoskeletal structures, the diffuse infiltration and hypertrophy of the masseter and medial pterygoid muscles, as well as the enlargement of the homolateral hemitongue and soft tissues of the cheek [4]. In a review of literature by Li et al. [15], yielding 59 patients, all patients exhibited a facial soft tissue mass characterized by nonpulsatile, noncompressible, and ill-defined features, resulting in facial asymmetry. Adipose tissue infiltration extended into the surrounding tissues, leading to muscle involvement in 24 cases (41%), encompassing muscles such as the masseters, pterygoid plate, and temporalis muscles, as well as affecting the parotid glands in 22 cases (37%). Oral malformations were also documented, with 19 cases (32%) featuring macroglossia, 19 cases (32%) showing early eruption of deciduous and permanent teeth on the affected side, 13 cases (22%) presenting with missing teeth, and 11 cases (19%) exhibiting macrodontism [15]. CIL-F stands apart from other forms of lipomatoses due to its impact on adjacent dentoskeletal structures [4]. It is known that the overgrowth of the osseous tissue by itself or following surgical intervention has the potential to cause exophytic bone formation and ankylosis of the TMJ [6]. The studies of Gupta et al. [2], Sahai et al. [6], and Keramidas et al. [7] describe CIL patients involving the face, wherein the disease is associated with underlying bony ankylosis of the temporomandibular joint. These studies presented patients similar to this case: diffuse right hemifacial fatty infiltration associated with TMJ involvement in the form of ankylosis, manifesting as severely restricted mouth opening. In all reported patients, surgical excision of the ankylosed mass was performed. Given the high recurrence rates, Sahai et al. [6] describe the essence of pre-operative recognition for operative management to not only release the ankylosis but also debulk the infiltrating lipomatosis. Dionne et al. [19] reported a postresection recurrence rate of 62.5%, which was reported during a follow-up period of 4 months to 20 years. Kang et al. [5] described an average of three debulking procedures per patient, with the tumor recurring in 9 of their 11 patients. Padwa et al. [20] described the role of growth hormones in recurrences and believe that any mass reduction before the end of adolescence is likely to fail due to recurrences.

Quinn et al. [21] reported ankylosed or resorbed joints with severe anatomic discrepancies as an indication of an alloplastic TMJ reconstruction. Wolford et al. [22] described 56 patients receiving patient-fitted TMJ total joint replacement devices. The 20-year follow-up study showed statistically significant improvements in incisal opening, jaw function, and joint pain, with 85.7% of the patients reporting significant improvement in their quality of life [22]. A TMJ prosthesis offers the advantages of immediate function, improvement in pain, the ability to correct bony defects and facial asymmetry, a lack of donor site morbidity, and a decreased length of hospital stay [21,22]. Based on orthopedic prosthesis experience, the expected lifespan should be at least 20 years [21]. The study by Wolford et al. concluded that after 19 to 24 years of service, the TMJ prosthesis continued to function well, and no prostheses were removed due to material wear [22]. Reasons for failure of the TMJ prosthesis were documented by Mercuri and Anspach [23] and include infection, component or fixation screw breakage, aseptic loosening, osteolysis, and periprosthetic bone fracture. Patients of a younger age undergoing primary alloplastic joint replacement have an elevated risk of revision [24]. Amarista et al. [25] reported that the primary reason for removal and replacement of the prosthesis was infection (21.1%), followed by 19.4% for an unspecified diagnosis (such as bone resorption, metallosis, or wear) and heterotopic ossification (15%). In this adult patient with a restricted mouth opening, a TMJ reconstruction was the treatment of choice, considering the risk of new CIL-F overgrowth or scar tissue development after gap-arthroplasty. A larger resection of the mandible was performed to create the largest possible gap, attempting to prevent reankylosis. Additionally, fat interposition was placed as much as possible for the same reason. Typically, TMJ replacement also aims for skeletal symmetry. Additional corrections can be incorporated into the TMJ design; supplementary orthognathic procedures can be performed if necessary; and occasionally, implants can be placed, all to achieve skeletal symmetry or correct skeletal asymmetries. However, these measures were not conducted in this case because there was a significant soft tissue asymmetry. Skeletal symmetry would not necessarily contribute to aesthetic improvement in this patient. In conventional cases, soft tissue corrections can be made to create symmetry, but this was not performed in this patient due to the unpredictable response of the soft tissues in the presence of his condition. In this case, the focus was solely on functional restoration without the additional pursuit of aesthetic correction through the creation of symmetry.

To our knowledge, no previous case of a TMJ reconstruction with a complete alloplastic total joint prosthesis in a CIL-F patient has been reported in the literature. After a one-year follow-up, no restriction of the mouth opening was evident; there were no longer difficulties with food intake, temporal facial pain was no longer present, and a stable occlusion was maintained. The TMJ prosthesis provided restoration of the function of the joint and led to an improvement in the patient’s quality of life. Further studies will need to be set up to investigate the long-term results of TMJ reconstruction in CIL-F patients.

## Data Availability

Data sharing not applicable.

## References

[1] Deshingkar S.A., Barpande S.R., Bhavthankar J.D. (2011). Congenital hemifacial hyperplasia. Contemp. Clin. Dent..

[2] Gupta R., Mukul S.K., Kumar P., Kumar A. (2020). Congenital infiltrating lipomatosis of the face with temporomandibular joint ankylosis. Natl. J. Maxillofac. Surg..

[3] Slavin S.A., Baker D.C., McCarthy J.G., Mufarrij A. (1983). Congenital infiltrating lipomatosis of the face: Clinicopathologic evaluation and treatment. Plast. Reconstr. Surg..

[4] Bouletreau P., Breton P., Freidel M. (2000). Congenital infiltrating lipomatosis of the face: Case report. Chang. Gung Med. J..

[5] Kang N., Ross D., Harrison D. (1998). Unilateral hypertrophy of the face associated with infiltrating lipomatosis. J. Oral Maxillofac. Surg..

[6] Sahai S., Rajan S., Singh N., Arora H. (2013). Congenital infiltrating lipomatosis of the face with exophytic temporomandibular joint ankylosis: A case report and review of the literature. Dentomaxillofacial Radiol..

[7] Keramidas T., Lagogiannis G., Vlachou V., Katsikeris N. (2012). Congenital infiltrating lipomatosis of the face with associated involvement of the TMJ structures. Case report and review of the literature. J. Cranio-Maxillofac. Surg..

[8] Yadav P., Roychoudhury A., Kumar R.D., Bhutia O., Bhutia T., Aggarwal B. (2021). Total Alloplastic Temporomandibular Joint Replacement. J. Maxillofac. Oral Surg..

[9] Nolte J.W., Alders M., Karssemakers L.H.E., Becking A.G., Hennekam R.C.M. (2020). Unilateral condylar hyperplasia in hemifacial hyperplasia, is there genetic proof of overgrowth?. Int. J. Oral Maxillofac. Surg..

[10] Kreutziger K.L. (1984). Surgery of the temporomandibular joint. I. Surgical anatomy and surgical incisions. Oral Surg. OralMed. Oral Pathol..

[11] Kramer T.T., Ho J.P.T.F., van de Vijfeijken S.E.C.M., Koutris M., van Riet T.C.T., de Lange J. (2022). Individueel vervaardigde prothesen voor ankylotische kaakgewrichten [Patient-specific joint prothesis for ankylotic temporomandibular joints]. Ned. Tijdschr. Voor Tandheelkd..

[12] van Ewijk L.J., van Riet T.C.T., van der Tol I.G.H., Ho J.P.T.F., Becking A.G. (2022). Power chains as an alternative to steel-wire ligatures in temporary maxillomandibular fixation: A pilot study. Int. J. Oral Maxillofac. Surg..

[13] Mercuri L.G., Ali F.A., Woolson R. (2008). Outcomes of total alloplastic replacement with periarticular autogenous fat grafting for management of reankylosis of the temporomandibular joint. J. Oral Maxillofac. Surg..

[14] Pires Fraga M.F., Mello D., Jorge D., Perin L.F., Helene A. (2009). Congenital infiltrating lipomatosis. J.Plast. Reconstr. Aesthetic Surg..

[15] Li Y., Chang G., Si L., Zhang H., Chang X., Chen Z., Huang J., Bai M., Wang Y., Long X. (2018). Congenital Infiltrating Lipomatosis of the Face: Case Report and Literature Review. Ann. Plast. Surg..

[16] Maclellan R.A., Luks V.L., Vivero M.P., Mulliken J.B., Zurakowski D., Padwa B.L., Warman M.L., Greene A.K., Kurek K.C. (2014). PIK3CA activating mutations in facial infiltrating lipomatosis. Plast. Reconstr. Surg..

[17] Unal O., Cirak B., Bekerecioglu M., Kutluhan A., Ugraş S., Tali T. (2000). Congenital infiltrating lipomatosis of the face with cerebral abnormalities. Eur. Radiol..

[18] Malik A., Jagmohan P., Thukral B.B., Khanna G., Rajni (2004). Congenital infiltrating lipomatosis of the face and neck. Acta Radiol..

[19] Dionne G.P., Seemayer T.A. (1974). Infiltrating lipomas and angiolipomas revisited. Cancer.

[20] Padwa B.L., Mulliken J.B. (2001). Facial infiltrating lipomatosis. Plast. Reconstr. Surg..

[21] Quinn P.D. (2000). Lorenz prosthesis. Oral Maxillofac. Surg. Clin. N. Am..

[22] Wolford L.M., Mercuri L.G., Schneiderman E.D., Movahed R., Allen W. (2015). Twenty-year follow-up study on a patient-fitted temporomandibular joint prosthesis: The Techmedica/TMJ Concepts device. J. Oral Maxillofac. Surg..

[23] Mercuri L.G., Anspach I.E. (2003). Principles for the revision of total alloplastic TMJ prostheses. Int. J. Oral Maxillofac. Surg..

[24] Ong K.L., Lau E., Suggs J., Kurtz S.M., Manley M.T. (2010). Risk of subsequent revision after primary and revision total joint arthroplasty. Clin. Orthop. Relat. Res..

[25] Amarista F.J., Mercuri L.G., Perez D. (2020). Temporomandibular Joint Prosthesis Revision and/or Replacement Survey and Review of the Literature. J. Oral Maxillofac. Surg..

